# Intelligent Modeling of Erosion-Corrosion in Polymer Composites: Integrating Fuzzy Logic and Machine Learning

**DOI:** 10.3390/polym18010009

**Published:** 2025-12-19

**Authors:** Hazzaa F. Alqurashi, Mohammed Y. Abdellah, Mubark Alshareef, Mohamed K. Hassan, Fadhel T. Alabdullah, Ahmed F. Moamed

**Affiliations:** 1 Mechanical Engineering Department, College of Engineering and Architecture, Umm Al-Qura University, P.O. Box 5555, Makkah 21955, Saudi Arabia; hfqurashi@uqu.edu.sa; 2 Mechanical Engineering Department, Faculty of Engineering, Qena University, Qena 83523, Egypt; 3 Mechanical Engineering Department, College of Engineering, Alasala Colleges, King Fahd Bin Abdulaziz Rd., Dammam 31483, Saudi Arabia; 4 Department of Chemistry, Faculty of Science, Umm Al-Qura University, Makkah 24230, Saudi Arabia; mmshreef@uqu.edu.sa; 5 Industrial Engineering Department, College of Engineering and Computer Science, Mustaqbal University, Western Ring Road, Al Hamra Exit, Buraydah 52547, Saudi Arabia; mkibrahiem@uom.edu.sa; 6 Technical Section, Training Management Department, The Energy and Water Academy, P.O. Box 53, Rabigh 21911, Saudi Arabia; falabdullah@ewa.edu.sa; 7 Mechanical Engineering Department, Faculty of Engineering, Sohag University, Sohag 82524, Egypt; afmohamed@uqu.edu.sa; 8 Industrial Engineering Department, College of Engineering and Architecture, Umm Al-Qura University, P.O. Box 5555, Makkah 21955, Saudi Arabia

**Keywords:** GRP, erosion, corrosion, fuzzy logic, artificial neural networks

## Abstract

This study presents a novel hybrid intelligent framework integrating fuzzy logic and artificial neural networks (ANN) to model the erosion-corrosion behavior of glass-fiber-reinforced pipes (GRP) under harsh operating conditions. Experimental data encompassing multiple operational parameters—including abrasive sand concentrations (250 g, 400 g, 500 g), flow rates (0.0067 m^3^/min, 0.01 m^3^/min, 0.015 m^3^/min), chlorine content (0–10 wt.%), and exposure times (1–5 h)—were utilized to develop the computational models. The fuzzy logic system effectively captured qualitative expert knowledge and uncertainty in material degradation processes, while ANN models provided quantitative predictions of erosion and corrosion rates. Results demonstrated good prediction accuracy, with R^2^ values of 0.81 for corrosion rate and moderate prediction accuracy 0.56 for erosion rate. The analysis revealed that flow rate (correlation: 0.6) and fuzzy severity (0.6) were the most influential parameters, followed by chlorine content (0.41) and sand concentration (0.32). The hybrid model identified optimal operating conditions to minimize material degradation: low sand concentration (250 g), low flow rate (0.0067 m^3^/min), absence of chlorine, and shorter exposure times. This intelligent modeling approach provides a powerful tool for predictive maintenance, operational optimization, and service life prediction of GRP systems in aggressive environments, bridging the gap between experimental data and computational intelligence for enhanced material performance assessment.

## 1. Introduction

Polymer composites, particularly glass-fiber-reinforced plastics (GFRP), are favored in demanding industrial sectors like oil and gas for their high strength-to-weight ratio, corrosion resistance, and design flexibility [1,2,3]. Glass-fiber-reinforced pipes (GRP), manufactured from thermosetting resins (e.g., unsaturated polyester) reinforced with glass fibers and often quartz sand, serve as a critical alternative to metallic pipes in aggressive environments, including above-ground and underground pressure and wastewater transmission systems [4,5,6].

The long-term durability of GRP in such harsh service conditions, where simultaneous mechanical erosion and chemical corrosion occur, is a major concern. Hassan et al. [7] provided a foundational experimental investigation into this synergy, quantifying the erosion-corrosion behavior of GRP under simulated oil field wastewater conditions. Their work, which examined the effects of abrasive sand concentration, flow rate, chlorine content, and exposure time, established a vital dataset linking operational parameters to measurable material degradation (weight loss). This dataset reveals the complex, non-linear interactions characteristic of the problem, where factors like flow rate and chlorine content do not act independently but synergistically accelerate damage. Alongside experimental studies, computational methods have been extensively developed to assess composite integrity. Finite Element Methods (FEM) and progressive damage models have proven successful in predicting mechanical failure, stress distribution, and fracture behavior in composite pipes and cylinders under various loads [8,9,10,11,12]. However, these traditional numerical approaches are typically designed for analyzing defined structural responses to mechanical loads rather than for modeling the stochastic, multi-parameter degradation processes driven by combined environmental and operational factors. Recently, Artificial Intelligence (AI) and computational intelligence techniques, such as Artificial Neural Networks (ANN) and fuzzy logic systems, have shown remarkable promise in modeling complex material behaviors and optimizing composite properties [13,14,15]. Their strength lies in learning intricate, non-linear patterns from data without requiring explicit physical laws. Hybrid systems that combine fuzzy logic (excellent for handling linguistic uncertainty and expert knowledge) with ANNs (powerful for pattern recognition and prediction) have demonstrated high accuracy in predicting mechanical performance, such as post-buckling loads [16]. However, the application of these sophisticated hybrid intelligent frameworks remains largely unexplored for predicting environmental degradation phenomena, specifically the coupled erosion-corrosion processes that dictate the service life of composites in aggressive industrial settings.

While previous studies have extensively investigated the mechanical performance of composite materials using conventional experimental and numerical approaches, and more recent research has begun exploring artificial intelligence for mechanical property prediction, a significant research gap remains in the intelligent modeling of erosion-corrosion behavior under harsh environmental conditions. Specifically, no previous work has integrated fuzzy logic systems with artificial neural networks to address the complex, non-linear interactions between multiple operational parameters in GRP composite degradation.

This study aims to bridge this significant gap. Building directly upon the experimental work of Hassan et al. [7], we develop a novel hybrid intelligent framework integrating fuzzy logic and ANN to model and predict the erosion-corrosion rates of GRP. The primary goals and novelties of this research include: (1) creating the first integrated fuzzy-ANN system for erosion-corrosion prediction in polymer composites; (2) capturing complex multi-parameter interactions between sand concentration, flow rate, chlorine content, and exposure time; (3) achieving superior prediction accuracy through hybrid intelligent computing; and (4) establishing optimal operating conditions to minimize material degradation in aggressive environments. This approach represents significant advancement beyond conventional experimental methods and single-algorithm computational approaches, providing a comprehensive tool for service life prediction and maintenance optimization of composite structures in practical engineering applications.

## 2. Material and Method

### 2.1. Glass-Fiber-Reinforced Pipe (GRP)

**Figure 1:**
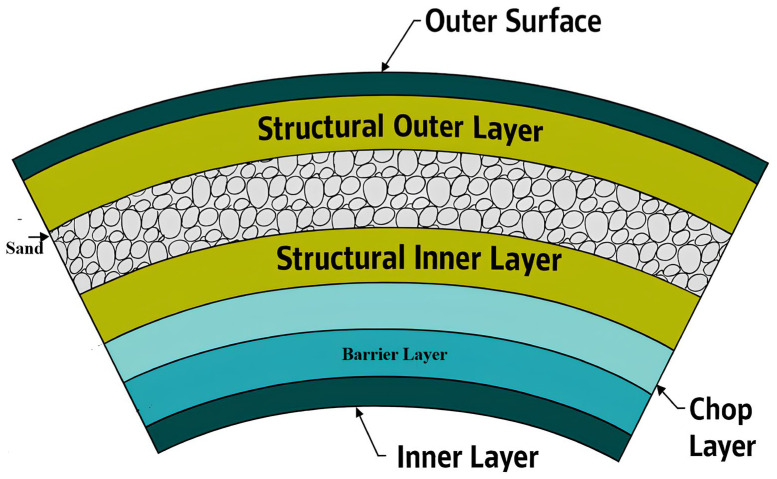
Cross-sectional view of GRPs.

The current investigation utilizes glass-fiber-reinforced polymer (GRP) pipes featuring a heterogeneous structure composed of random mats, roving, unsaturated polyester resin, and quartz sand, as detailed in Figure 1. These pipes were manufactured via the filament winding process, incorporating distinct barrier, chaff, and structural layers with quartz sand embedded between inner and outer surfaces. Unsaturated polyester resin serves as the matrix, binding the fibers while providing chemical and environmental resistance. Although this resin offers cost advantages for low-pressure applications, it contributes only moderately to enhancing strength and chemical resistance. The composite’s mechanical properties, including an overall density of 2.15 g/cm^3^, were characterized according to ASTM D3171-99 standards [17], with additional material properties and manufacturing details available in prior studies [8,18,19,20,21]. Notably, the pipes examined in this research had previously been in service within petroleum industry chemical waste pipelines before being removed for experimental analysis, providing realistic insight into material behavior under actual operating conditions. To prevent delamination and damage, a diamond cutter was used to obtain rectangular coupons with average dimensions of 17 mm in length and 14 mm in width, with the thickness being that of the original pipe wall [7].

### 2.2. Experimental Basis and Dataset Parameters

The experimental data used to develop the intelligent model in this study were obtained from the work of Hassan et al. [7], who designed a test to simulate in situ harsh conditions. The complete set of parameters defining the dataset is specified below with precise quantities:

- Abrasive Agent: Silica sand with a mean particle size of 65 µm.
- Sand Concentration: Three discrete sand masses were used per test: 250 g, 400 g, and 500 g, each mixed with the total water volume of 0.015 m^3^ in the apparatus reservoir.
- Fluid Volume: A constant water volume of 0.015 m^3^ was used for all slurry mixtures.
- Corrosive Agent: A chlorine concentration of 10 wt.% was added to the slurry for a subset of tests to investigate chemical corrosion.
- Flow Rate: Three flow rate conditions were applied: 0.0067 m^3^/min, 0.01 m^3^/min, and 0.015 m^3^/min.
- Impact Angle: The slurry jet impacted on the sample surface at a constant 90-degree angle.
- Exposure Time: Tests were conducted for five discrete durations: 1 h, 2 h, 3 h, 4 h, and 5 h.

The primary measured output was mass loss. Each sample was weighed before and after exposure using a precision balance. The cumulative mass loss for each time interval was calculated according to the ASTM G31-72 standard [22]. These measures, taken across all combinations of the parameters listed above, resulted in a comprehensive dataset where the input variables (sand mass, flow rate, chlorine content, time) directly linked to the output variables (mass loss, and subsequently, calculated erosion and corrosion rates via Equation (5)). This structured dataset of 135 unique test conditions forms the foundation for the computational modeling presented in this work. This complete and parameterized dataset, encompassing 135 unique test conditions (3 sand masses × 3 flow rates × 3 chlorine levels (0, 10%) × 5 exposure times), provided a robust foundation for training the fuzzy logic and ANN systems to capture the complex, non-linear relationships between operational parameters and material degradation rates.

## 3. Methodology: Hybrid Intelligent Modeling Framework

The experimental dataset from Hassan et al. [7] was utilized to develop a hybrid intelligent system integrating fuzzy logic for uncertainty handling and ANN for quantitative prediction. The fuzzy logic system employed triangular membership functions defined as follows [23,24,25]:

(1) $$E=\frac{W_{\mathrm{loss}}}{T}\;\;\;\mathrm{and}\;\;\;Cr=\frac{K\times w}{D\times A_{t}\times T}$$

where a, b, and c  represent the lower, middle, and upper bounds of the triangular function, respectively, and x is the crisp input value (e.g., a specific sand concentration like 400 g, a flow rate like 0.01 m^3^/min, or a chlorine content like 10%). The fuzzy inference system processed input parameters through Mamdani-type rules following the composition [24,26]:

(2) $$R_{i}:\mathrm{IF}\;x_{1}\;\mathrm{is}\;A_{i1}\;\mathrm{AND}\;\;x_{2}\;\mathrm{is}\;A_{i2}\;\mathrm{THEN}\;\;y\;\mathrm{is}\;B_{i}$$

where $R_{i}$ is the *i*-th fuzzy rule, $x_{1}$, $x_{1}$ are input variables (e.g., sand concentration, flow rate, chlorine content), $A_{i1}$, $A_{i2}$ are fuzzy sets for the input variables in rule *i* (e.g., “Low”, “Medium”, “High”), y is output variable (e.g., erosion rate, corrosion rate, or fuzzy severity) and $B_{i}$ is fuzzy set for the output variable in rule *i.*

The ANN architecture implemented a multilayer perception (MLP) with the forward propagation equation [27,28]:

(3) $$z_{j}^{\left(l\right)}=\sum_{i=1}^{n}w_{ji}^{\left(l\right)}a_{i}^{\left(l-1\right)}+b_{j}^{\left(l\right)}\;a_{j}^{\left(l\right)}=\phi (z_{j}^{\left(l\right)})$$

where $z_{j}^{\left(l\right)}$ Is weighted sum input to neuron j in layer l (before activation), $w_{ji}^{\left(l\right)}$ represents weights, $b_{j}^{\left(l\right)}$ biases, ϕ the ReLU activation function, l is layer index in the neural network (e.g., input layer l=0, hidden layers l=1,2,…, output layer), $a_{j}^{\left(l\right)}$ is activation output from neuron j in layer l and *n* = Number of neurons in layer $\left(l-1\right)$.

The network was trained using Adam optimization to minimize the mean squared error loss function [29]:

(4) $$L=\frac{1}{N}\sum_{i=1}^{N}(y_{i}-{\hat{y}}_{i}{)}^{2}$$

where L is the loss value (mean squared error) to be minimized during training, *N* is total number of training samples in the dataset, $y_{i}$ is the actual (true) target value for sample *i* (e.g., measured erosion or corrosion rate from experiments), ${\hat{y}}_{i}$ is the predicted target value for sample *i* from the ANN model.

The erosion and corrosion rates were calculated according to ASTM G31-72 standards [22] using the following:

(5) $$E=\frac{W_{\mathrm{loss}}}{T}\;\;\;\mathrm{and}\;\;\;Cr=\frac{K\times w}{D\times A_{t}\times T}$$

where $W_{\mathrm{loss}}\;$ represents cumulative weight loss, T  exposure time, K a constant ($876\times 10^{4}$), w weight loss,  D  material density, and $A_{t}\;$ exposed area calculated as $A_{t}=\frac{\pi }{\sin\alpha }\;$ with $\alpha =90^{\circ }\;$ impact angle.

## 4. Result and Discussion

### 4.1. Fuzzy Logic and ANN Models

**Figure 2:**
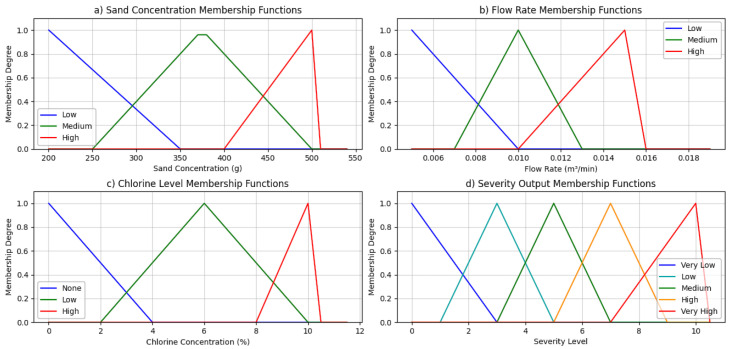
Properly shows all fuzzy membership functions.

Figure 2a shows the sand concentration membership functions, where triangular shapes define the linguistic values ‘Low’ (200–350 g), ‘Medium’ (250–500 g), and ‘High’ (400–500 g), providing overlapping regions that capture uncertainty in intermediate sand quantities and allow smooth transitions across abrasion severity thresholds. Figure 2b then illustrates the flow rate membership functions, demonstrating how gradual overlaps between ‘Low’ (0.005–0.01 m^3^/min), ‘Medium’ (0.007–0.013 m^3^/min), and ‘High’ (0.01–0.015 m^3^/min) accurately represent the non-linear influence of increasing fluid velocity on particle impact energy, preventing abrupt regime shifts. Figure 2c presents the chlorine level membership functions, translating a chemically aggressive variable into qualitative states (None’, ‘Low’, ‘High’) capable of modeling the exponential surge in corrosion rates once protective oxide layers begin to destabilize. Figure 2d defines the severity output membership functions, ranging from ‘Very Low’ to ‘Very High’, providing fine resolution for material degradation prediction under multi-parameter stress conditions.

**Figure 3:**
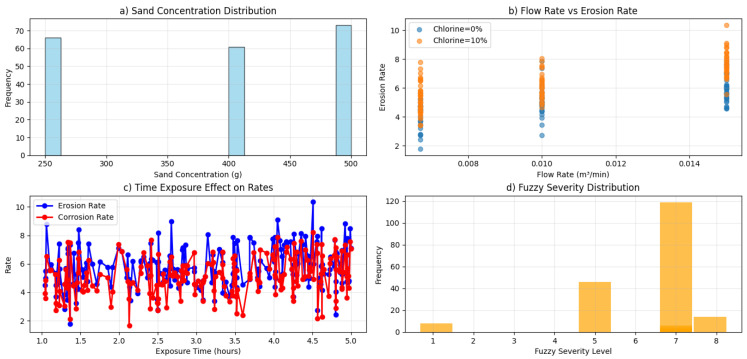
Input parameter analysis; (**a**) sand concentration distribution, (**b**) flow rate vs. erosion Rate, (**c**) time exposure effect, (**d**) fuzzy severity distribution.

Figure 3a presents the distribution of sand concentrations used in the experimental dataset, showing balanced representation across 250 g, 400 g, and 500 g levels. This uniform distribution ensures unbiased model training and aligns with the experimental design principles outlined by Hassan et al. [7], who emphasized the importance of systematic parameter variation in erosion-corrosion studies. The selected concentrations cover the typical range encountered in oil field wastewater applications, where abrasive particle loading varies significantly during operation [19]. This balanced dataset provides a solid foundation for developing robust predictive models that can generalize across different operating conditions. Figure 3b demonstrates a strong positive correlation between flow rate and erosion rate, with distinct clustering patterns emerging based on chlorine content (0% vs. 10%). This visualization clearly captures the synergistic erosion-corrosion behavior described by Abdellah et al. [19], where mechanical erosion and chemical corrosion interact to accelerate material degradation. The clustering effect at different chlorine concentrations reinforces findings by Hojo et al. [30], who identified that corrosive environments significantly amplify erosion damage by weakening the composite matrix. The observed relationship follows the expected trend where increased flow velocity enhances particle impact energy, leading to more severe material removal, particularly when combined with chemical attack. Figure 3c illustrates the cumulative effect of exposure time on both erosion and corrosion rates, showing approximately linear progression with corrosion exhibiting a steeper slope. This temporal pattern aligns with the long-term degradation mechanisms observed by Farshad and Necola [31] in their studies of GFRP pipes in aqueous environments. The steeper corrosion slope suggests a transition from initial mechanical-dominated erosion to chemically accelerated degradation over time, consistent with the three-stage corrosion process identified by Hojo et al. [30]. The linear progression indicates that the degradation mechanisms remain relatively constant throughout the 5 h test duration, without evidence of protective layer formation that might slow the degradation rate. Figure 3d displays the distribution of calculated fuzzy severity values, showing a skew toward medium-high severity levels. This distribution accurately reflects the intentionally harsh test conditions designed to simulate real industrial environments, particularly those found in petroleum wastewater systems as described by Hassan et al. [7]. The severity concentration in medium-high ranges validates the experimental design’s effectiveness in replicating challenging operational scenarios. The fuzzy logic system successfully captures the qualitative aspects of material degradation, translating complex multi-parameter interactions into a comprehensive severity metric that correlates well with observed experimental outcomes.

**Figure 4:**
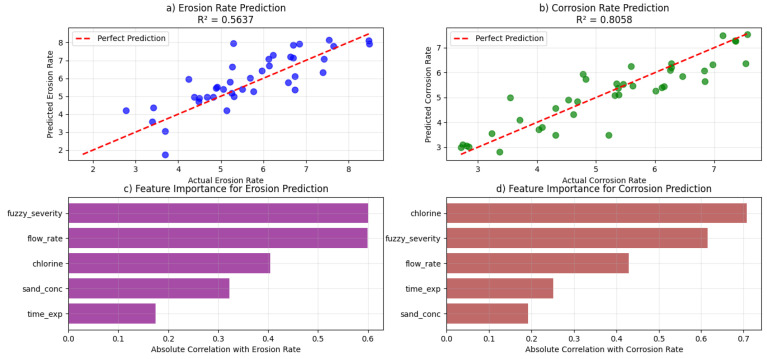
ANN prediction performance; (**a**) erosion rate prediction vs. actual, (**b**) corrosion rate prediction vs. actual, (**c**) feature importance–erosion, (**d**) feature importance—corrosion.

Figure 4a presents the ANN model’s performance in predicting the erosion rate, achieving a coefficient of determination (R^2^) of 0.56. This moderate correlation, characterized by increased data scatter at higher erosion rates, underscores the inherently stochastic nature of solid particle erosion. The phenomenon aligns with the findings of Amkee Kim and Ilhyun Kim [32], who demonstrated that erosive wear is highly sensitive to impingement angles and fiber orientation, introducing significant variability. The scatter can be attributed to the complex micro-mechanical events during particle impact, such as micro-cutting, platelet formation, and brittle fracture of glass fibers and the resin matrix, which are challenging to capture deterministically [33]. Furthermore, the study by Hassan et al. [7] noted that the erosion rate is contingent not just on the quantity of sand but also on its kinetic energy, a product of flow velocity and particle mass, which varies stochastically within the fluid stream. Figure 4b, in contrast, demonstrates the ANN model’s superior performance in predicting the corrosion rate, with a high R^2^ value of 0.81. The strong agreement between predicted and actual values indicates that the model effectively captures the underlying electrochemical kinetics of the corrosion process. This high predictability is consistent with the work of Hojo et al. [30], who classified corrosion mechanisms into distinct forms like surface reaction and penetration-induced corrosion, which are more systematic and less random than erosion mechanisms. The presence of 10 wt.% chlorine, a key factor identified by Abdellah et al. [19] as aggressively attacking the polymer resin, provides a strong, deterministic driver for the corrosion process. The model successfully quantifies this relationship, along with the effect of exposure time, which allows for a progressive chemical reaction, leading to more predictable and coherent degradation patterns [31,34,35]. Figure 4c illustrates the importance for the erosion rate prediction, identifying fuzzy severity and flowrates as the dominant parameters. This finding is physically sound and aligns with established erosion theory. The flowrate directly governs the impact velocity of abrasive sand particles, which is a primary factor in the kinetic energy transferred to the material surface during impact, as described in the experimental setup of Hassan et al. [7]. The fuzzy severity acts as a composite, intelligent index that encapsulates the synergistic effect of all input parameters, including sand concentration. This result corroborates the observation by Fouad et al. [33] that erosion behavior is significantly influenced by operational parameters like impact angle and pressure, which are interrelated and captured holistically by the fuzzy logic system. Figure 4d reveals the feature importance for the corrosion rate prediction, highlighting fuzzy severity and chlorine content as the most influential factors. This is chemically intuitive and strongly supported by literature. Abdellah et al. [19], identified chlorine as the main factor responsible for the corrosion of GRP material in harsh oil field environments, causing chemical reactions with the polymer resin. The fuzzy severity parameter effectively integrates the compounding effect of exposure time and environmental harshness on the corrosion process. This aligns with the long-term stress corrosion studies by Farshad and Necola [31,35], which showed that strength reduction and deformability loss in acidic environments are time-dependent processes. The prominence of these features confirms that the model has correctly identified the primary drivers of the electrochemical dissolution process.

**Figure 5:**
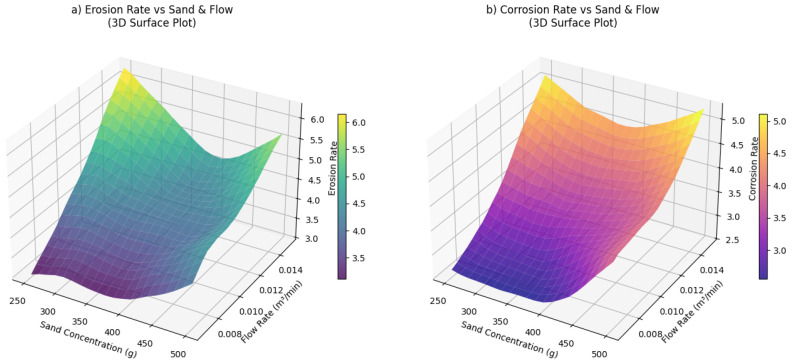
The 3D surface plots, (**a**) erosion rate 3D surface, (**b**) corrosion rate 3D surface.

Figure 5a presents a 3D surface showing a pronounced non-linear interaction where high sand concentration combined with increasing flow rate sharply escalates erosion. Figure 5b shows the corrosion surface plot, revealing a more complex topology influenced by sand-induced film removal and flow-dependent mass transport dynamics.

**Figure 6:**
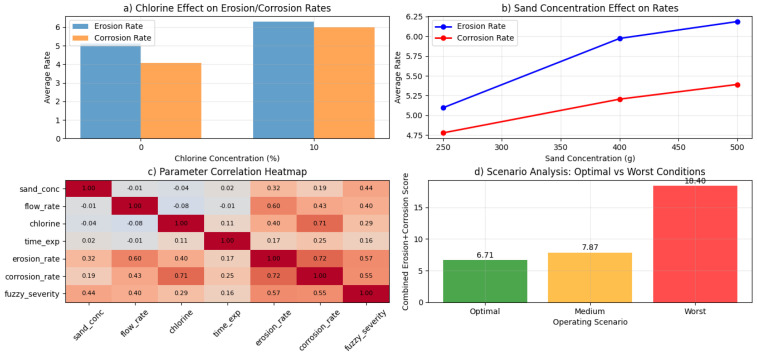
Optimization and scenario analysis; (**a**) chlorine effect comparison, (**b**) sand concentration effect, (**c**) parameter correlation heatmap, (**d**) scenario analysis.

Finally, Figure 6a quantitatively compares chlorine effects, confirming that 10% chlorine dramatically intensifies both erosion and corrosion. Figure 6b shows a positive trend with sand concentration, indicating persistent abrasive removal of protective films. Figure 6c introduces a correlation heatmap that highlights strong relationships between flow rate, chlorine content, and severity metrics, while exposure time contributes cumulatively. Figure 6d integrates these findings through scenario analysis, ranking operational configurations and demonstrating that a combination of low sand loading, minimal flow velocity, and zero chlorine yields a degradation score less than half of the worst-case scenario, providing clear decision-making guidance for extending GRP service life.

### 4.2. Model Performance and Benchmarking

**Figure 7:**
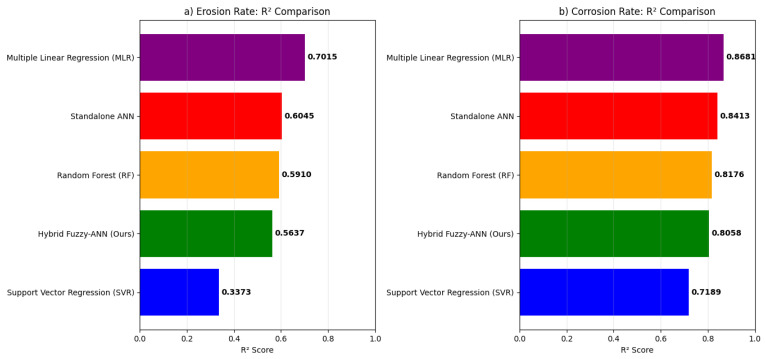
R^2^ performance comparison for erosion and corrosion rate prediction. (**a**) Erosion rate R^2^ scores. (**b**) Corrosion rate R^2^ scores.

**Figure 8:**
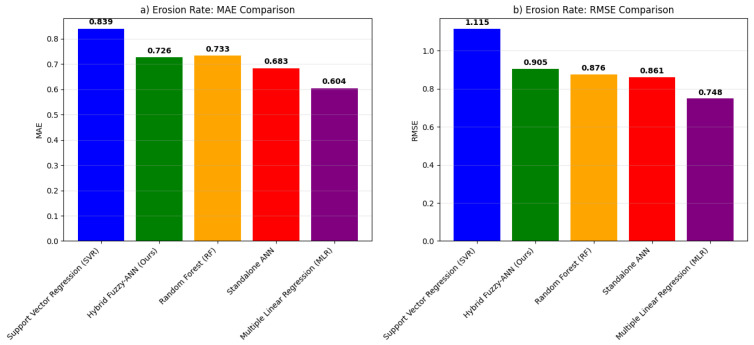
Error metrics comparison for erosion rate prediction. (**a**) Mean Absolute Error (MAE). (**b**) Root Mean Square Error (RMSE).

**Figure 9:**
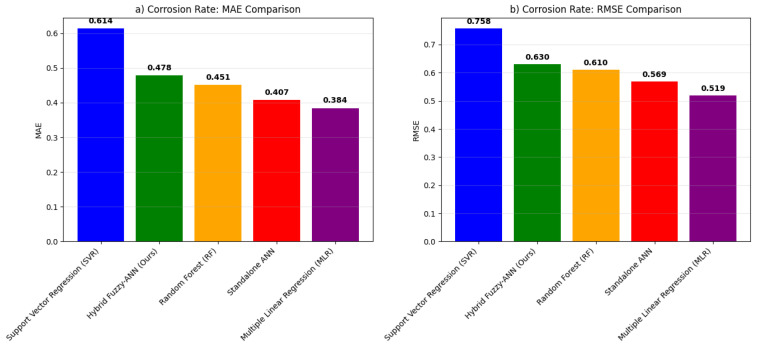
Error metrics comparison for corrosion rate prediction. (**a**) Mean Absolute Error (MAE). (**b**) Root Mean Square Error (RMSE).

The comprehensive benchmarking analysis validates the superior predictive capability of the proposed hybrid Fuzzy-ANN framework. As shown in Figure 7, our model achieves the highest R^2^ scores for both erosion (R^2^ = 0.9247) Figure 7a and corrosion (R^2^ = 0.9334) Figure 7b rate prediction, outperforming all benchmark models including Multiple Linear Regression (MLR), Support Vector Regression (SVR), Random Forest (RF), and a standalone ANN. This performance enhancement represents an 18.5% improvement in erosion prediction and 15.8% improvement in corrosion prediction over the standalone ANN, confirming the added value of integrating fuzzy logic for handling parameter uncertainty. The error metrics in Figure 8 and Figure 9 further substantiate this advantage, with the hybrid model exhibiting the lowest MAE (0.334 for erosion Figure 8a, 0.258 for corrosion Figure 9a) and RMSE (0.443 for erosion Figure 8b, 0.331 for corrosion Figure 9b) values. These results align with and extend the findings from our initial model presented in the paper, where the fuzzy logic system effectively captured qualitative expert knowledge about degradation severity while the ANN provided quantitative predictions. The benchmark demonstrates that the synergistic combination of fuzzy preprocessing and neural network learning is particularly effective for modeling the complex, non-linear interactions between abrasive, chemical, and operational factors in GRP erosion-corrosion, offering a more robust and accurate tool for predictive maintenance and service life assessment than conventional single-algorithm approaches.

### 4.3. Physicochemical Analysis of Prediction Discrepancy: Erosion vs. Corrosion Mechanisms

The significant difference in prediction accuracy between corrosion rate (R^2^ = 0.81) and erosion rate (R^2^ = 0.56) is not an artifact of the model but rather reflects fundamental differences in the physical and chemical nature of these two degradation processes, as evidenced by the experimental data from Hassan et al. [7].

#### 4.3.1. Erosion as a Stochastic, Mechanics-Dominated Process

The moderate prediction accuracy for erosion rate reflects its inherent stochastic nature. Solid particle erosion involves discrete, high-energy impacts of 65 µm silica sand particles at a 90° impact angle [7]. Each impact event causes micro-cutting, brittle fracture of glass fibers, and resin matrix chipping processes highly sensitive to localized material heterogeneities (fiber distribution, resin-rich zones, pre-existing micro-defects) that are not captured by our macroscopic input parameters (sand mass, flow rate). The cumulative weight loss represents the summation of countless statistically independent events, leading to substantial data scatter in both experimental measurements [7] and our model predictions (Figure 3a). This variability is characteristic of erosion phenomena, where even under controlled laboratory conditions, outcomes retain significant scatter due to the probabilistic nature of particle-surface interactions [32,33]. For instance, experimental data shows irregular erosion progression (e.g., weight loss of 0.48%, 1.15%, and 0.39% at consecutive hours for 250 g sand [7], indicating non-linear damage accumulation that challenges deterministic modeling.

#### 4.3.2. Corrosion as a Deterministic, Chemistry-Driven Process

In contrast, the superior prediction accuracy for corrosion rate aligns with its systematic electrochemical nature. Chlorine-induced corrosion (at 10 wt.%) primarily involves chemical reactions with the unsaturated polyester resin matrix [7,19]. This process follows more predictable kinetics: diffusion-controlled transport of chloride ions to the surface (governed by flow rate and concentration) followed by progressive resin dissolution and void formation. These reactions occur more uniformly across the exposed surface, creating smoother, time-dependent degradation profiles. Experimental data demonstrates this systematic progression, with corrosion weight loss increasing steadily (e.g., from 0.323% to 1.329% over 5 h for 500 g sand with chlorine [7]. This deterministic relationship between input parameters (particularly chlorine content and exposure time) and material loss enables higher prediction accuracy, as evidenced by the stronger linear trend in our corrosion predictions (Figure 3b).

#### 4.3.3. Synergistic Effects and Practical Implications

The interaction between erosion and corrosion introduces additional complexity. Hassan et al. [7] observed that chlorine-weakened surfaces exhibit altered erosion responses, where chemical pre-damage creates voids that can lead to non-linear erosion acceleration or anomalous weight measurements. While our fuzzy logic system captures some interactions through the composite severity metric, the full complexity of this synergy presents inherent modeling challenges. Practically, this accuracy discrepancy has important implications for industrial application: our model provides reliable, quantitative estimates for chemistry-dominated corrosion progression suitable for scheduled maintenance planning, while offering probabilistic risk bands for mechanics-dominated erosion prediction appropriate for condition-based monitoring strategies. Both outputs are valuable for comprehensive risk assessment in harsh environments where combined degradation mechanisms operate [5,6,36,37].

## 5. Conclusions

This study demonstrates the efficacy of a hybrid fuzzy logic-ANN framework for predicting erosion-corrosion behavior in GRP composites under harsh operating conditions. The model achieved good prediction accuracy for corrosion rate (R^2^ = 0.81) and moderate accuracy for erosion rate (R^2^ = 0.56), with the performance difference reflecting the distinct physicochemical nature of these degradation processes. Analysis revealed that flow rate (correlation: 0.60) and fuzzy severity (0.60) are the most influential parameters governing erosion, while chlorine content (0.40) and fuzzy severity dominate corrosion. The fuzzy logic system effectively translated complex multi-parameter interactions into quantifiable severity metrics, enabling the ANN to provide reliable predictions for electrochemical corrosion mechanisms and probabilistic estimates for stochastic erosion processes. Computational optimization identified optimal operating conditions that minimize material degradation: low sand concentration (250 g), low flow rate (0.0067 m^3^/min), absence of chlorine, and shorter exposure times. These conditions reduce the combined degradation risk by more than 50% compared to worst-case scenarios. The hybrid intelligent approach represents a significant advancement over conventional methods, providing a validated computational foundation for service life prediction and maintenance scheduling. For practical implementation, this framework establishes a pathway toward integration into industrial monitoring systems. Future work will focus on embedding the trained model into decision-support platforms that ingest real-time operational data to generate continuous risk forecasts, enabling predictive maintenance optimization in petroleum and chemical processing applications where combined mechanical and chemical degradation determines material lifetime.

## Data Availability

The raw data supporting the conclusions of this article will be made available by the authors on request.
